# Intermittent oroesophageal vs. nasogastric tube feeding in frail elderly with dysphagia: a retrospective comparative study

**DOI:** 10.3389/fmed.2026.1867115

**Published:** 2026-07-28

**Authors:** Ying Xu, Li Liu, Dan Zhao, Nan Shao, Xiaohui Li, Ying Wang, Bo Li

**Affiliations:** 1 Department of Geriatric, Dalian Municipal Friendship Hospital, Dalian, Liaoning, China; 2 Endoscopy Center, Dalian Municipal Friendship Hospital, Dalian, Liaoning, China; 3 Department of Nursing, Dalian Municipal Friendship Hospital, Dalian, Liaoning, China

**Keywords:** dysphagia, frail elderly, intermittent oroesophageal feeding, nasogastric tube feeding, nutritional support

## Abstract

**Background:**

Dysphagia affects 15%−20% of community elderly, 50%−75 % of nursing-home and >80 % of hospitalized patients; 60 % cases stem from neurologic disease. We compared intermittent oro-esophageal (IOE) vs. nasogastric tube (NGT) feeding in 120 frail dysphagic elders (Jun–Dec 2024).

**Methods:**

The sample size was calculated based on the expected prevalence of aspiration pneumonia (25 vs. 8%), with 60 participants enrolled per group. Frail elderly patients with dysphagia were randomly allocated to the intermittent oro-esophageal or nasogastric tube group using a random number table. Statistical analyses were performed using SPSS 26.0, including *t*-tests, chi-square tests, and multivariate logistic regression, with *P* < 0.05 considered significant.

**Results:**

Baseline comparable. Both groups improved albumin and swallowing equally, yet IOE cut aspiration pneumonia from 23.3 % (NGT) to 8.3 %, enhanced oral intake and MNA-SF without added mucosal injury. Frailty, age, stroke history and NGT predicted complications; Kubota grade-3 patients gained most from IOE.

**Conclusion:**

IOE is safer and more effective than NGT for frail elderly dysphagia, reducing pneumonia and restoring oral feeding.

## Introduction

With the accelerating global aging population, swallowing dysfunction in frail elderly has become a major unmet health challenge in clinical practice (1–3). Dysphagia is highly prevalent among the elderly: its prevalence reaches 15%−20% in community-dwelling elderly, rises to 50%−75% in nursing home residents, and exceeds 80% in hospitalized elderly patients (4–6). The etiologies are complex and diverse: 60% of cases are caused by neurological diseases such as stroke and Parkinson's disease (7, 8). When combined with age-related physiological degeneration of swallowing, such as weakened pharyngeal contraction, delayed laryngeal elevation, this significantly increases the risk of adverse outcomes (9, 10). More notably, there is a bidirectional pathological relationship between dysphagia and frailty—insufficient intake of protein and nutrients due to swallowing difficulties accelerates sarcopenia, while reduced oropharyngeal muscle strength caused by frailty further worsens swallowing function (11–13). This ultimately forms a malnutrition-dysphagia-frailty vicious cycle. 45%−60% of patients develop malnutrition, and 70% of elderly pneumonia cases are directly associated with aspiration (14, 15). Therefore, nutritional support regimens for this population must simultaneously address three goals: nutrient supply, swallowing function protection, and complication prevention.

Frailty is an age-related clinical syndrome characterized by decreased physiological reserve and multisystem dysfunction, which results in increased vulnerability to stressors, impaired stress resistance, and elevated risks of adverse health outcomes (16). An individual is defined as frail if they meet three or more of the following criteria: unintentional weight loss, low grip strength, exhaustion, slow walking speed, and low physical activity level (17, 18). Such as Unintentional weight loss ≥5% in the past 1 year, Low grip strength: < 26 kg for men and < 18 kg for women, measured by a handheld dynamometer, Self-reported exhaustion, and slow walking speed: ≤ 0.8 m/s, Low physical activity level. The main adverse consequences, including increased risk of aspiration pneumonia, malnutrition, dehydration, prolonged hospital stay, reduced quality of life, and elevated mortality in this vulnerable elderly population.

Currently, the mainstream clinical enteral nutrition support method is indwelling nasogastric tube (NGT) feeding (19–21). While it can solve short-term feeding problems, it has significant limitations in frail elderly (22, 23). Long-term NGT indwelling damages the physiological barriers of the nasal cavity and digestive tract, causing local complications such as esophageal mucosal injury and oral infections (24, 25). Long-term nasogastric tube (NGT) feeding as continuous indwelling for ≥28 days. And adverse effects include aspiration pneumonia, esophageal mucosal injury, and upper gastrointestinal complications, with relevant literature references added accordingly. More critically, it cannot avoid the risk of gastric content reflux, leading to persistently high prevalence of aspiration pneumonia (26–28). Clinical cases show that vomiting and reflux caused by gastric motility disorders in neurology patients using NGT significantly increase aspiration risk, posing a life-threatening threat especially to elderly patients with impaired airway protection barriers (29, 30). Although gastrostomy can reduce aspiration risk, its invasive nature—along with complications like stoma infection and surgical trauma—makes it poorly suited for the tolerance of frail elderly patients (31, 32). These drawbacks highlight the inadequacy of traditional tube feeding methods in balancing association and safety, underscoring the urgent need to explore nutritional support models more aligned with the physiological characteristics of frail elderly individuals (33, 34).

As a novel enteral nutrition technology, intermittent oroesophageal feeding (IOE) offers a new solution to the above dilemmas through its intermittent operation of tube insertion during feeding and removal after feeding (35). Unlike long-term NGT indwelling, IOE reduces continuous catheter stimulation to the digestive tract mucosa, lowering the risk of mucosal injury and infection (36). Meanwhile, the stimulation of the oropharynx during intubation can trigger swallowing reflexes, endowing IOE with dual value in nutritional support and swallowing function rehabilitation (37, 38). China has issued the Technical Requirements for Intermittent Oroesophageal Feeding in Adults, which clearly includes dysphagia caused by multiple etiologies such as frailty and Parkinson's disease within its scope of application, confirming its clinical applicability (39). However, existing studies mostly focus on patients with stroke as the sole etiology, lacking systematic research on the special population of frail elderly (40). In particular, there is a scarcity of head-to-head comparative data between IOE and NGT regarding improvements in nutritional indicators, swallowing function recovery, and complication rates (41). Furthermore, the underlying mechanism linking nutrition, swallowing, and frailty under IOE intervention has not been thoroughly explored.

This study conducted a retrospective analysis focusing on elderly patients with debilitating dysphagia, comparing the clinical differences between IOE and NGT. Objective nutritional indicators, such as body mass index (BMI), serum albumin (ALB) and subjective scores, such as Mini Nutritional Assessment-Short Form (MNA-SF) were included (42). Swallowing function was evaluated using the Kubota Water Swallowing Test and Functional Oral Intake Scale (FOIS). Frailty status was quantified via the Fried Frailty Scale. Complications and safety indicators, such as aspiration pneumonia, esophageal mucosal injury were systematically monitored. This study aims to provide evidence-based basis for selecting nutritional support regimens for frail elderly with dysphagia and optimize clinical comprehensive management strategies.

## Materials and methods

### Basis for sample size calculation

The sample size of this study was calculated based on the prevalence of aspiration pneumonia (the primary outcome indicator). Referring to previous similar studies, the prevalence of aspiration pneumonia was approximately 25% in the NGT group and 8% in the IOE group. With α set at 0.05 and β at 0.2, PASS 15.0 software was used for calculation, indicating a minimum of 52 cases required per group. Considering a 15% attrition rate, 60 cases were finally included in each group, resulting in a total sample size of 120 cases.

### Sample collection and inclusion/exclusion criteria

This study was approved by the Medical Ethics Committee of Dalian Friendship Hospital (approval number: LL-2024-018-02). From June 2024 to December 2024, frail elderly with dysphagia hospitalized in the Geriatrics Department, Rehabilitation Department, and Neurology Department of our hospital were consecutively enrolled. Tube feeding operations were performed by trained and qualified medical staff. Eligible patients were included via consecutive sampling and divided into the IOE group and NGT group using a random number table.

### Inclusion criteria

1. Aged ≥65 years;

2. Meeting the Fried Frailty Syndrome criteria (≥3 out of 5 items);

3. Moderate-to-severe dysphagia confirmed by Kubota Water Swallowing Test Grade 3–5 (energy intake < 60% of recommended amount);

4. MNA-SF score ≤ 11 or Nutrition Risk Screening 2002 (NRS-2002) score ≥3, requiring enteral nutrition;

5. Normal digestive function and expected survival ≥3 months;

6. Informed consent signed by the patient or legal representative.

### Exclusion criteria

1. Absolute contraindications to IOE/NGT (e.g., esophageal obstruction, history of perforation);

2. Complications such as severe hepatic/renal failure or acute infectious shock, or absolute contraindications to enteral nutrition;

3. Inability to cooperate with assessments (e.g., mini-mental state examination [MMSE] score < 10);

4. Receipt of parenteral nutrition, swallowing rehabilitation training, or participation in other studies within 2 weeks before enrollment;

5. Refusal to sign informed consent or high risk of loss to follow-up.

### Data types and description methods

#### Continuous data

Presented as mean ± standard deviation (x ± s) if normally distributed, and as median (interquartile range) [M (Q1, Q3)] if not normally distributed.

#### Categorical data

Presented as case number (percentage, *n*, %). Ordinal data (e.g., Kubota Water Swallowing Test scores, FOIS scores): presented as case number (percentage, *n*, %) or median.

### Statistical tests and model setting

#### Baseline data comparison

Independent samples *t*-test for continuous data, chi-square test for categorical data, and Kruskal–Wallis *H*-test for ordinal data.

#### Correlation analysis

Pearson correlation analysis was used for normally distributed continuous data (e.g., correlation between ALB and Kubota scores, blood glucose fluctuations and MNA-SF scores), with correlation coefficient r calculated.

#### Stratified analysis

One-way analysis of variance (ANOVA) was used to analyze the association between baseline Kubota grade and FOIS improvement, comparing differences between groups.

#### Multivariate regression model

Multivariate logistic regression analysis was used to explore the association between Fried Frailty Scale scores and aspiration pneumonia, as well as the association between age/comorbidities and complications. Independent variables included age, history of stroke, feeding method, and Fried score; the dependent variable was set as “complication occurrence = 1” and “no complication = 0”. Variables were screened using the stepwise method (entry criterion α = 0.05, elimination criterion α = 0.10). Model fit: evaluated via the Hosmer–Lemeshow test (*P* > 0.05 indicated good fit). Statistical software: all analyses were performed using SPSS 26.0. A *P*-value < 0.05 was considered statistically significant.

## Results

### No atatistically significant differences in baseline data between IOE and NGT groups

A total of 120 frail elderly with dysphagia were enrolled, with 60 cases in both the IOE group and NGT group. There were no significant differences between the two groups in demographic characteristics (age, gender, disease duration, history of stroke), nutritional indicators (BMI, ALB, prealbumin [PAB], hemoglobin [Hb], MNA-SF score), swallowing function indicators (Kubota Water Swallowing Test grades, FOIS score), frailty indicators (total Fried score, prevalence of five Fried sub-items), and laboratory parameters (blood glucose, electrolytes, liver/kidney function) (Table 1, *P* > 0.05). This indicated balanced and comparable baseline characteristics between the two groups, eliminating the impact of baseline confounding factors on intervention effects.

**Table 1:** Comparison of baseline data between patients of IOE and NGT. (x ± s or *n*, %).

Group	IOE group (*n* = 60)	NGT group(*n* = 60)	*P*
Age (years)	78.2 ± 6.5	77.8 ± 6.3	0.782
Gender (*n*)			0.725
Male	32	34	
Female	28	26	
Duration of illness (months)	3.5 ± 1.8	3.7 ± 1.6	0.516
History of stroke(*n*, %)	38 (63.3%)	40 (66.7%)	0.689
BMI ((kg/m^2^)	21.3 ± 2.4	21.1 ± 2.5	0.673
Serum albumin (ALB, g/L)	31.2 ± 3.5	30.8 ± 3.7	0.568
Prealbumin (PAB, mg/L)	178.5 ± 25.3	175.2 ± 26.8	0.472
Hemoglobin (Hb, g/L)	118.6 ± 12.5	116.8 ± 13.2	0.451
(MNA-SF) score	8.6 ± 2.1	8.3 ± 2.2	0.439
Water swallowing test (*n*, %)			0.815
Level 1	2 (3.3%)	1 (1.7%)	
Level 2	15 (25.0%)	14 (23.3%)	
Level 3	21 (35.0%)	22 (36.7%)	
Level 4	18 (30.0%)	19 (31.7%)	
Level 5	4 (6.7%)	4 (6.7%)	
FOIS score	3.2 ± 1.0	3.1 ± 1.1	0.628
Fried score	3.2 ± 1.1	3.3 ± 1.0	0.654
Weight loss (*n*, %)	35 (58.3%)	37 (61.7%)	0.682
Fatigue (*n*, %)	42 (70.0%)	43 (71.7%)	0.835
Grip strength decline (*n*, %)	39 (65.0%)	41 (68.3%)	0.695
Slowed walking speed (*n*, %)	45 (75.0%)	46 (76.7%)	0.821
Decreased activity (*n*, %)	48 (80.0%)	47 (78.3%)	0.813
Blood glucose (mmol/L)	6.5 ± 1.3	6.7 ± 1.2	0.418
Serum potassium (mmol/L)	3.8 ± 0.3	3.7 ± 0.4	0.176
Serum sodium (mmol/L)	138.5 ± 2.6	137.9 ± 2.8	0.253
Serum creatinine (mmol/L)	88.6 ± 15.2	90.3 ± 14.8	0.542
Blood urea nitrogen (mmol/L)	6.8 ± 1.5	7.0 ± 1.4	0.479
ALT (U/L)	32.5 ± 8.6	33.8 ± 8.2	0.412
AST, (U/L)	35.2 ± 9.1	36.5 ± 8.7	0.435

### Nutritional improvement promoted swallowing function recovery in both groups, with no difference in association

After intervention, serum ALB levels significantly increased from baseline in both groups (IOE group: 31.2–35.8 g/L; NGT group: 30.8–34.9 g/L), while Kubota Water Swallowing Test scores significantly decreased (indicating improved swallowing function). Pearson correlation analysis showed a positive correlation between ALB levels and Kubota scores in both groups (IOE group: *r* = 0.42, *P* = 0.001; NGT group: *r* = 0.39, *P* = 0.002), suggesting that nutritional improvement promoted swallowing function recovery regardless of IOE or NGT use. Further inter-group comparison showed no statistically significant difference in correlation coefficients between the two groups (*r* difference = 0.03, *P* = 0.815), indicating no difference in the association between nutrition and swallowing between the two feeding methods (Table 2).

**Table 2:** Correlation between serum albumin (ALB) and Wada drinking water test score.

Group	Sample size (*n*)	Post-intervention ALB level (g/L, x ±s)	Post-intervention water swallowing test score (levels, x ±s)	Correlation coefficient (*r*)	*P*–value
IOE Group	60	35.8 ± 3.2	2.1 ± 0.9	0.42	0.001
NGT Group	60	34.9 ± 3.4	2.3 ± 1.0	0.39	0.002
Between-group comparison	–	–	–	0.03	0.815

### Lower prevalence of aspiration pneumonia in the IOE group; frailty severity was an independent risk factor for complications

During the intervention period, the prevalence of aspiration pneumonia in the IOE group (8.3%, 5/60) was significantly lower than that in the NGT group (23.3%, 14/60). Multivariate logistic regression analysis showed that after adjusting for age and history of stroke, the risk of aspiration pneumonia in the NGT group was 3.25 times that of the IOE group (OR = 3.25, 95% CI: 1.58–6.68, *P* = 0.002). Meanwhile, each 1-point increase in the Fried Frailty Scale score significantly increased the risk of aspiration pneumonia by 89% (OR = 1.89, 95% CI: 1.21–2.95, *P* = 0.005), confirming that frailty severity was an independent risk factor for complications in the IOE group (Table 3).

**Table 3:** Correlation between fried frailty scale score and prevalence of aspiration pneumonia.

Group	Prevalence rate (*n*, %)	Odds ratio (OR)	95% confidence interval (CI)	*P*–value
IOE group (reference value)	5 (8.3%)	1	–	–
NGT group	14 (23.3%)	3.25	1.58–6.68	0.002
For each 1-point increase	–	1.89	1.21–2.95	0.005

### No difference in esophageal mucosal injury prevalence between groups; IOE group showed better oral feeding ability

There was no significant difference in the prevalence of esophageal mucosal injury between the IOE group and NGT group (10.0 vs. 11.7%, *P* = 0.783). Pearson correlation analysis showed no significant association between esophageal mucosal injury and FOIS scores in either group (IOE group: *r* = −0.12, *P* = 0.352; NGT group: *r* = −0.15, *P* = 0.268), suggesting that local mucosal adverse reactions did not affect the recovery of oral feeding ability. Further comparison of post-intervention FOIS scores showed that the IOE group (5.8 ± 1.1 points) had a significantly higher score than the NGT group (4.5 ± 1.0 points, *P* < 0.001), indicating that IOE had advantages in improving oral feeding ability without increasing the risk of esophageal mucosal injury (Table 4).

**Table 4:** Correlation between esophageal mucosal injury and oral feeding ability score (FOIS).

Analysis Content	IOE group (*n* = 60)	NGT group (*n* = 60)	*P*-value
Prevalence rate of esophageal mucosal injury (*n*, %)	6 (10.0%)	7 (11.7%)	0.783
Correlation coefficient (*r*) between injury and FOIS	−0.12		0.352
		−0.15	0.268
Post-intervention FOIS score (points, x ± s)	5.8 ± 1.1	4.5 ± 1.0	<0.001

### No difference in blood glucose fluctuations between groups; IOE group showed more significant nutritional improvement

There was no significant difference in blood glucose fluctuation amplitude between the two groups during the intervention (IOE group: 1.8 ± 0.5 mmol/L vs. NGT group: 1.9 ± 0.6 mmol/L, *P* = 0.385). Additionally, no significant association was found between blood glucose fluctuations and MNA-SF scores in either group (IOE group: *r* = −0.08, *P* = 0.542; NGT group: *r* = −0.11, *P* = 0.416), suggesting that both feeding methods had consistent effects on blood glucose stability, and blood glucose fluctuations did not interfere with nutritional improvement. After intervention, the MNA-SF score of the IOE group (11.2 ± 2.3 points) was significantly higher than that of the NGT group (9.5 ± 2.1 points, *P* < 0.001), confirming that IOE was more effective in improving nutritional status (Table 5).

**Table 5:** Correlation between blood glucose fluctuation amplitude and MNA-SF nutritional score.

Analysis content	IOE group (*n* = 60)	NGT group (*n* = 60)	Statistical method	*P*-value
Prevalence rate of esophageal mucosal injury (*n*, %)	6 (10.0%)	7 (11.7%)	Chi-square test	0.783
Correlation coefficient (*r*) between injury and FOIS	−0.12	–	Pearson correlation analysis	0.352
	–	−0.15		0.268
Post-intervention FOIS score (points, x ± s)	5.8 ± 1.1	4.5 ± 1.0	Independent samples *t*-test	<0.001

### Patients with baseline Kubota grade 3 were the optimal population for IOE intervention

Stratified analysis showed that among patients with baseline Kubota Grade 3, the FOIS improvement value in the IOE group (2.8 ± 0.7 points) was significantly higher than that in the NGT group (1.6 ± 0.6 points, *P* < 0.001). However, no significant differences in FOIS improvement values were observed between the two groups in patients with baseline Kubota Grade 1, 2, 4, or 5 (all *P* > 0.05). This indicated that baseline Kubota Grade 3 was the optimal population for IOE intervention, as these patients experienced more significant recovery of oral feeding ability after IOE intervention (Table 6).

**Table 6:** Correlation between baseline Wada drinking water test scores and FOIS scores at 3 months after intervention.

Baseline water swallowing test level	Total sample size (*n*)	IOE group FOIS increase (points, x ±s)	NGT group FOIS increase (points, x ±s)	*P*-value (between groups)
Level 1	3	1.0 ± 0.0	0.9 ± 0.1	0.612
Level 2	29	2.2 ± 0.6	1.9 ± 0.5	0.085
Level 3	43	2.8 ± 0.7	1.6 ± 0.6	< 0.001
Level 4	37	1.9 ± 0.5	1.5 ± 0.4	0.052
Level 5	8	1.2 ± 0.4	1.0 ± 0.3	0.391

### Age, history of stroke, NGT feeding, and frailty were independent risk factors for complications

A risk prediction model constructed via multivariate logistic regression showed that each 10-year increase in age (OR = 1.62, *P* = 0.006), history of stroke (OR = 2.18, *P* = 0.001), NGT feeding (OR = 2.89, *P* < 0.001), and each 1-point increase in Fried score (OR = 1.75, *P* = 0.002) were independent risk factors for complications. The model showed good fit (Hosmer–Lemeshow test *P* = 0.512, *R*^2^ = 0.38), which can be used in clinical practice to predict the risk of complications in frail elderly with dysphagia during tube feeding and provide reference for feeding method selection (Table 7).

**Table 7:** Correlation between age, underlying diseases, and prevalence of complications.

Predictor	Coding method	Regression coefficient (β)	Standard error (SE)	Odds ratio (OR)	95% CI	*P*-value
Age	For each 10-year increase	0.48	0.19	1.62	1.15–2.28	0.006
History of stroke	Yes = 1, No = 0	0.78	0.27	2.18	1.36–3.49	0.001
Feeding method	IOE = 0, NGT = 1	1.06	0.32	2.89	1.67–5.01	<0.001
Fried frailty score	For each 1-point increase	0.56	0.21	1.75	1.23–2.48	0.002

Model R^2^ = 0.38, Hosmer–Lemeshow test *P* = 0.512.

## Discussion

The findings of this study highlight the potential benefits of intermittent oroesophageal feeding (IOE) over traditional nasogastric tube (NGT) feeding in frail elderly with dysphagia (43). As the global population ages, the prevalence of dysphagia among the elderly is increasing, posing significant challenges to clinical practice (44, 45). Dysphagia not only affects nutrient intake but also exacerbates frailty, leading to a vicious cycle of malnutrition, dysphagia, and frailty. This study aimed to address this challenge by comparing the clinical association and safety of IOE and NGT feeding methods.

The results showed that both IOE and NGT feeding methods were effective in improving nutritional status and swallowing function. However, the IOE group had a significantly lower prevalence of aspiration pneumonia compared to the NGT group. This finding is crucial, as aspiration pneumonia is a common and severe complication in elderly patients with dysphagia (46). The lower prevalence of aspiration pneumonia in the IOE group suggests that IOE may be a safer alternative to NGT feeding, especially for patients with impaired airway protection mechanisms.

Moreover, the IOE group demonstrated better oral feeding ability, as evidenced by higher Functional Oral Intake Scale (FOIS) scores. This indicates that IOE not only provides adequate nutrition but also enhances the patients' ability to feed orally, which is essential for improving their quality of life (47). The study also found that IOE was more effective in improving nutritional status, as shown by higher Mini Nutritional Assessment-Short Form (MNA-SF) scores in the IOE group after intervention.

The study identified patients with baseline Kubota Grade 3 as the optimal population for IOE intervention. This suggests that IOE may be particularly beneficial for patients with moderate dysphagia, as they experienced more significant recovery of oral feeding ability. This finding is important for clinical practice, as it helps to identify the specific patient population that may benefit most from IOE. IOE does not require long-term tube indwelling, which eliminates continuous stimulation of the upper esophageal sphincter and significantly reduces gastroesophageal reflux and micro-aspiration. The tube is inserted only during feeding and removed immediately afterward, preserving laryngeal closure and natural airway protection during daily activities. The Kubota test is a brief bedside screening tool that mainly reflects obvious swallowing safety, while silent aspiration and reflux-related aspiration are the main causes of pneumonia in this population—these are not fully captured by Kubota scores. Therefore, the lower pneumonia rate reflects a reduction in reflux and persistent tube-related aspiration risk, rather than a difference in overt swallowing function. This explanation strengthens the clinical interpretation of our findings.

The study also constructed a risk prediction model, which identified age, history of stroke, NGT feeding, and frailty as independent risk factors for complications. This model can be used in clinical practice to predict the risk of complications in frail elderly with dysphagia during tube feeding and provide reference for feeding method selection. We acknowledge that this was a retrospective study without rater blinding, which may introduce potential assessment bias. We clarify that the Kubota test is a bedside screening tool, not a definitive diagnostic measure, and cannot fully detect silent aspiration or subtle swallowing impairment. Kubota grade 3 subgroup: we critically discuss why these patients benefited most, and they retain sufficient laryngeal sensitivity, cough reflex, and partial swallowing function, making them more responsive to IOE stimulation. Patients with too mild or too severe dysphagia gain less obvious advantage. We have revised the conclusion to avoid overstatement. Instead of claiming strong evidence, we now state that IOE shows favorable safety and efficacy and may be a preferred option for frail elderly patients with dysphagia, acknowledging that several outcomes did not differ significantly between groups.

In summary, this study provides evidence that IOE is a more effective and safer nutritional support method for frail elderly with dysphagia compared to NGT feeding. IOE not only improves nutritional status and swallowing function but also reduces the risk of aspiration pneumonia and enhances oral feeding ability. These findings suggest that IOE should be considered as a preferred feeding method for this vulnerable population. Future research should focus on exploring the underlying mechanisms linking nutrition, swallowing, and frailty under IOE intervention and further validating the findings in larger and more diverse patient populations.

## Conclusion

In conclusion, our study demonstrates that IOE was associated with more favorable outcomes in this study for frail elderly with dysphagia compared to nasogastric tube (NGT) feeding, as it significantly reduces the prevalence of aspiration pneumonia while enhancing oral feeding ability. IOE also proves to be more effective in improving the overall nutritional status of patients. These findings suggest that IOE may be considered as an alternative in selected frail elderly patients over NGT in clinical practice for this specific patient population to optimize their health outcomes.

## Data Availability

The original contributions presented in the study are included in the article/Supplementary material, further inquiries can be directed to the corresponding author.
