# Supplementary material for: Intermittent oroesophageal vs. nasogastric tube feeding in frail elderly with dysphagia: a retrospective comparative study
Source: Front Med (Lausanne). 2026 Jul 28;13:1867115. doi: 10.3389/fmed.2026.1867115 (PMC13457138; doi:10.3389/fmed.2026.1867115)
Supplement: Supplementary file 1 [file Table_1.DOCX]

Ethical review opinions

Document No.: AF / SW-07 / 2023-01.0

Approval letter of Dalian Friendship Hospital: LL-2024-018-02

project name

Clinical application of intermittent oral tube feeding in senile patients with swallowing disorder

100 Taste

The second and third families of senile diseases

Principal investigator

Xu Ying

professional ranks and titles

co-chief superintendent nurse

The type of research

Prospective, observational study

Project source

import

Source of funds

Other: Peak plan

Bid unit

Dalian Friendship Hospital

Group leader unit

Dalian Friendship Hospital

Main participating units

Dalian Friendship Hospital

audit-review file

1. List of documents submitted for review: 2. Table of review and amendment details: 3. Research Plan (2.0 / 2024.8.1)

Review category

□ Initial Review □ Review □ Amendment Review □ Annual / Periodic Review □ Serious Adverse Event Review □ Deviation protocol review □ Suspension / Terminate Study Review □ Study Completion Review

Review method

□ Meeting review □ Summary review □ Emergency meeting review

Review decision

| Approval □ modified approved □ modified □ retrial not approved □ suspension or termination of study □ submitted for joint trial

Review opinions

Drug Regulatory Administration / National Health Commission of drug clinical trial quality management standard (2003), according to the National Health Commission involving human life science and medical research ethics review (2023), national ethical review of drug clinical trials (2010), clinical trials of medical devices (2022), clinical in vitro diagnostic reagents. After the initial review of the project (2021), WMA Declaration of Helsinki and the EC ethics Committee, the EC approved the EC to conduct clinical study in our hospital in accordance with the approved study protocol and other documents. Then, clinical studies were carried out in strict accordance with the research protocol approved by the ethics committee to protect the health and rights of the subjects. Please follow the Measures for ethical Review of Life Science and Medical Research Involving People (2023) and GCP and other regulations

Frequency of the agreed annual / periodic follow-up reviews

□ 3 months □ 6 months □ 12 months

Whether to adjust the frequency of the agreed follow-up review

□ Adjustment (adjustment to _____) □ No adjustment □ is not applicable

Validity of consent letter

1. The validity period of the Consent Letter is 1 year; 2. The validity date of the validity letter: From May 22,2024 to May 22,2025; 3. Please submit the annual / periodic study progress report one month before the expiration date (April 2025) to apply for extending the validity period of the consent letter.

contacts

Qin Yi

contact number

0411-82718822

Signature by the Chairman / Authorizer

VO

date

20216.5.2

Ethics Committee

The Ethics Committee of Dalian Friendship Hospital

For the project of "initial review", the researcher is requested to apply for the project registration account number and password of the project before the study starts. Register with the Medical Research Registration Information System. Items in other review categories are registered in the "system" after receiving the ethical review "consent". The multi-center research is filed by the team leader unit.
